# The Redefinition and Volumization of the Lip Area with Hyaluronic Acid: A Case Series

**DOI:** 10.3390/jcm13195705

**Published:** 2024-09-25

**Authors:** Nazaret Ruiz, Roberto Miranda Lopez, Rubén Marques, Silvia Fontenete

**Affiliations:** 1 Private Clinic, 33202 Gijón, Spain; 2 Hospital Cruz Roja, 33202 Gijón, Spain; doctormirandalopez@gmail.com; 3 Medical Department, BioScience GmbH, 28008 Madrid, Spain; ruben.marques@biosciencegmbh.com (R.M.); silviafontenete@gmail.com (S.F.); 4 Institute of Biomedicine (IBIOMED), University of Leon, 24071 León, Spain

**Keywords:** filler, hyaluronic acid, lips, perioral rejuvenation, redefinition, volumization

## Abstract

**Background**: The increasing popularity of non-surgical cosmetic enhancements for the lower face and perioral area, particularly through hyaluronic acid (HA) fillers, reflects the growing desire for improved lip volume and definition. This study showcases the effects of a specific HA filler on lip fullness, shape, and overall perioral rejuvenation. **Methods**: We conducted a retrospective single-site observational analysis of adult female patients treated with Genefill Soft Fill HA injections in the lips and perioral areas. Both patient and physician satisfaction were evaluated using the Likert scale and Global Aesthetic Improvement Scale (GAIS), respectively. The outcomes for natural appearance, volume, and durability were assessed using a five-point scale. The patients were followed up with for up to six months to monitor any adverse events. **Results**: The cohort included thirteen female patients with an average age of 55.3 ± 8.3 years. Approximately 1.2 ± 0.4 mL of filler was used per patient. The results indicate high satisfaction, with scores above 4 for naturalness, volume, and durability. Over 92% of patients reported a significant improvement in appearance. No moderate or severe adverse events were reported. **Conclusions**: Genefill Soft Fill HA filler is both effective and safe for enhancing lip esthetics, with high satisfaction rates among recipients and no significant adverse events observed.

## 1. Introduction

Both intrinsic (genetic) and extrinsic (environmental) factors play a crucial role in the phenotypic changes observed in the aging and photoaging of facial and perioral tissues [1]. Intrinsic aging is largely governed by genetic factors and hormonal variations, leading to the progressive breakdown of collagen and elastin fibers, adipose tissue, and other key skin components like hyaluronic acid (HA), which causes skin thinning, reduced elasticity, and wrinkle development [2,3]. In contrast, photoaging is primarily caused by prolonged exposure to ultraviolet (UV) radiation, which worsens these structural changes and speeds up visible signs of aging, including hyperpigmentation, uneven skin texture, and more pronounced wrinkles [4]. Moreover, oxidative stress and chronic inflammation are recognized as significant contributors to the pathogenesis of both aging processes as they drive cellular aging and promote ongoing tissue degradation [5].

The advancement of skin research and a deeper comprehension of skin features and anatomy have led to a significant increase in non-surgical esthetic treatments aimed at facial rejuvenation [6]. HA, calcium hydroxylapatite (CaHA), and polylactic acid (PLLA) are widely used as temporary fillers to enhance skin volume and reduce the appearance of wrinkles. These temporary fillers improve biocompatibility, longevity, and the ability to stimulate collagen production, offering more natural and lasting results in facial rejuvenation procedures [7,8]. Facial aging is accompanied by perioral hard and soft tissue changes, leading to wrinkles, folds, and loss of contour and volume in the lip. The lips and the perioral area are of remarkable importance in a youthful appearance, attractiveness, and beauty [9,10]. Therefore, these age-related changes in labial features can significantly impact an individual’s emotional well-being, social interactions, and self-perceived happiness [11]. The use of HA filler in the lips is usually requested more by aging patients; however, this trend has been changing, and more younger patients are requesting this treatment. The treatment in aging patients is related to restoring or accenting the natural curves of the lip and reflating the lip to minimize vermillion creasing, along with correcting rhytids in the white lip skin. The augmentation of the lips in younger patients requires higher volumes, particularly in the vermillion, to enhance lip size and shape more noticeably [12]. A deep understanding of facial anatomy and the common changes that occur with aging and strategic esthetic planning are critical for achieving results that appear natural [13]. Conversely, exceeding the reasonable expectations of treatments can lead to outcomes that look artificial or even cause tissue damage and complications [14,15]. Lip treatments with HA fillers are regarded as safe when performed by an experienced provider using proper techniques and the appropriate type of filler [16]. However, with the growing demand for this treatment, there has been a rise in reported cases of temporary complications in the literature [17,18,19,20]. Although typically mild and transient, complications can be minimized through careful patient selection, precise injection techniques, and a thorough understanding of facial anatomy, which are essential for reducing risks, preventing filler migration, and ensuring optimal outcomes [16,18,21].

Moreover, the success of lip augmentation is influenced by various factors, including the choice of HA filler, the volume injected, the frequency of follow-up treatments, the injection technique utilized, the degree of cross-linking in the HA product, the patient’s skin type, the practitioner’s experience, and the method of evaluation [5].

The commercially available filler Genefill Soft Fill ^®^ (BioScience GmbH, Dümmer, Germany) is characterized by its combination of cross-linked HA with a non-crosslinked HA and its low BDDE content, which is below 0.01 ppm.

This case series aimed to confirm the beneficial effects of the Genefill Soft Fill (improved esthetics with no adverse effects) for lip augmentation and contouring.

## 2. Methods

This retrospective observational case series study, conducted in Gijón, Spain, analyzed patients who received HA dermal filler (Genefill Soft Fill; BioScience GmbH, Germany) for lip augmentation and contouring. The participants were treated as part of routine clinical practice at esthetic clinics. The data were drawn from information already recorded in the database.

Thirteen older patients underwent lip augmentation and contouring and perioral wrinkle filling with HA filler (GeneFill Soft Fill) from June 2022 to October 2022 with complete medical records for up to 6 months. All patients were treated by a single experienced plastic surgeon with extensive expertise in dermal fillers.

This study was conducted in accordance with the principles of the Declaration of Helsinki and the International Conference on Harmonization Good Clinical Practice guidelines. All patients were informed about the product and procedure and signed an informed consent form, allowing for their anonymized clinical data to be used for future training and scientific purposes.

Due to the non-interventional and observational nature of this study and in accordance with Law 14/2007 of 3 July on Biomedical Research, EU Regulation 2016/679 of the European Parliament and of the Council, 04/2016, and Organic Law 3/2018 of 5 December on Personal Data Protection and the Guarantee of Digital Rights (LOPDGDD), this study did not require authorization from a Research Ethics Committee since it does not have an impact on personal data protection or the integrity of the subjects.

### 2.1. Hyaluronic Acid Filler

Genefill Soft Fill is an HA filler from BioScience GmbH composed of 20 mg/mL of cross-linked HA and 2 mg of non-crosslinked HA from a non-animal source. Genefill Soft Fill is indicated for general lip rejuvenation, hydration and definition of the lips, and harmonious volumization of the lips or asymmetry correction.

### 2.2. Patients

As per routine clinical practice, the exclusion criteria for injection or for lip augmentation treatment included a known allergy to Genefill Soft Fill filler components, an immunocompromised status, coagulopathy, an active infection, inflammation, and malignancy of the neck. Patients who were pregnant and breastfeeding were also not treated with GeneFill Soft Fill.

### 2.3. Injection Technique

As per routine clinical practice, each treatment was performed in an outpatient setting, lasting approximately 10 to 15 min. Before injection, the overlying skin was cleansed of makeup, and topical anesthetic cream composed of lidocaine and prilocaine was applied for approximately 5 min. A topical antiseptic chlorhexidine solution was applied to sterilize the skin.

**Figure 1:**
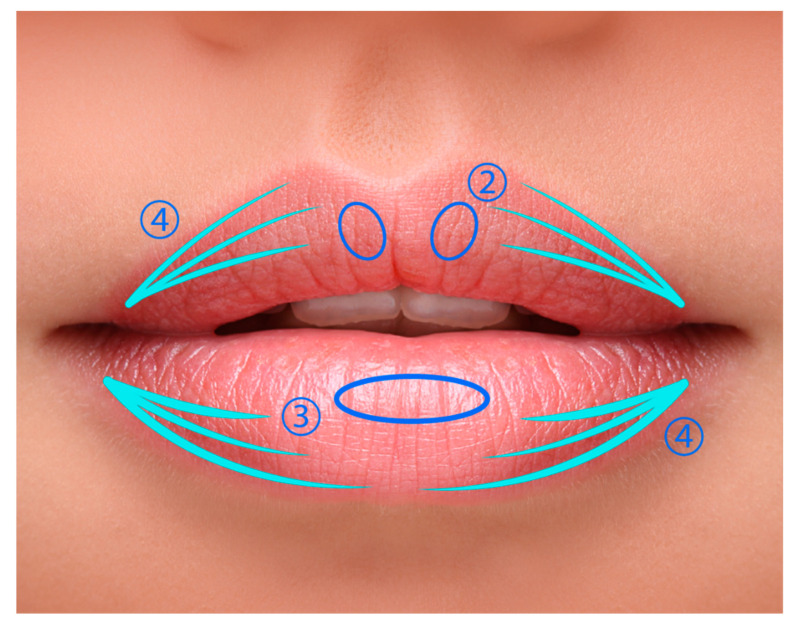
The distribution of HA dermal filler volume in the lips: (1) 0.1–0.2 cc per side; (2) 0.05–0.1 cc per side; (3) 0.1–0.2 cc total; and (4) 0.01 cc per line, shown in light blue. This figure was personally designed by the authors to accurately represent the procedure described.

Deep tissue injections were administered at the mucosal transition from dry to wet using a 27 and 30 G needle placed intramuscularly. Specific targeting of the tubercles in the central lower lip was achieved with deep injections using a 30 G needle from the skin–mucosa transition. Additionally, surface contouring was performed by executing retrograde injections along the lip profile and into the superficial layers of the dry mucosa with a 30 G needle to enhance definition. The approach and distribution of HA filler is described in Figure 1.

At the perioral lines, HA filler was injected into the subcutaneous tissue using a linear threading technique and a 27 G needle.

To reduce the risk of intravascular injection, care was taken to identify facial arteries, located either at the labial commissure or following the contour of the philtrum, ensuring the precise placement of HA filler in the lips.

### 2.4. Evaluation of Treatment Experience and Outcomes

Patient demographic data, including gender and age, were recorded. Details of each session, including the number and date and the volume of HA injected were also collected.

Syringe force (using a 27 G needle) was evaluated on a scale ranging from 1 (minimum force) to 5 (maximum force).

Natural results, volumizing effect, and durability were evaluated by the physician on a 5-point scale, where 1 is the minimum score and 5 is the maximum score. The investigator took photographs at every visit (before the injection; after the injection; and at week 2, weeks 4 and 3, and 6 months) of the treated area to evaluate the performance of the treatment at each visit compared to the before-treatment photographs. Aesthetic improvement was evaluated at 1 month following treatment, with comparisons made to the pretreatment baseline. The assessment was conducted by the physician using the Global Aesthetic Improvement Scale (GAIS), a standardized tool for evaluating cosmetic outcomes. The GAIS rates improvement on a scale from 1 to 5, where 1 represents a “Very Much Improved” and optimal result, 2 indicates “Much Improved” with noticeable enhancement but potential for slight improvement with a touch-up, 3 reflects “Improved” with clear changes but the need for re-treatment, 4 signifies “No Change” from the original condition, and 5 denotes that the appearance has “Worsened”.

Patient satisfaction was measured using a Likert scale. This scale ranges from 1 to 5, where 1 corresponds to “Very Dissatisfied,” 2 to “Dissatisfied”, 3 to “Slightly Satisfied”, 4 to “Satisfied”, and 5 to “Very Satisfied”.

Possible adverse events (such as bruising, edema, redness, itching, inflammation, etc.) were recorded immediately after the intervention and at various time points until the end of the follow-up.

A 6-point modified Visual Analog Scale (VAS) was used to assess the patients’ pain levels immediately after injection. A combined Visual Analog Scale (VAS) with corresponding facial expressions to represent different levels of pain intensity (Wong–Baker FACES Pain Rating Scale). Each face reflects the severity of pain ranging from “No Pain” to “Worst Pain Possible”.

Patients rated their pain on a scale from 0 to 5, where 0 indicates “no pain” and 5 represents the “maximum pain experienced”. The scale is defined as follows: 0 indicates the absence of pain; 1 represents mild pain; 2 describes moderate pain; 3 corresponds to severe pain; 4 represents very severe pain; and 5 indicates the worse pain possible.

In the assessment of swelling, a 5-point scale was used to categorize the severity of the swelling 48 h after the treatment. The scale is defined as follows: 1 indicates the absence of swelling; 2 represents subjective swelling that is not noticeable to others; 3 describes mild to moderate swelling that is resolved within 24 h; 4 corresponds to moderate swelling that does not double the immediate post-treatment volume and persists for more than 24 h; and 5 represents severe swelling that doubles the immediate post-treatment volume and lasts for more than 24 h. Moreover, swelling and other possible adverse events were assessed by the physician during follow-up visits through visual inspection. Additionally, before and after photographs, taken at consistent angles and lighting conditions, were used to track changes over time.

### 2.5. Data Analysis

In this study, descriptive statistical analyses were conducted to summarize the data. The measures of central tendency and dispersion, such as mean and standard deviation (SD), were calculated to describe continuous variables. For categorical variables, percentages were used to represent the distribution of scores across different groups. All analyses were performed using standard statistical software (GraphPad Prism 10), and the results are presented as the mean ± SD for continuous data and as percentages (%) for categorical data to provide a clear understanding of the distribution patterns in the sample.

Non-parametric Kruskal–Wallis test was employed to compare the differences in scores across multiple follow-up time points (1 day, 2 weeks, 4 weeks, 3 months, and 6 months). This test was chosen due to the ordinal nature of the satisfaction scores and the presence of non-normally distributed data. When a significant difference was detected, Dunn’s post hoc test was performed to identify which specific time points showed significant pairwise differences. Dunn’s test was adjusted for multiple comparisons using the Bonferroni correction to control for type I errors. A *p*-value of less than 0.05 was considered statistically significant.

## 3. Results

### 3.1. Baseline Demographic Characteristics

**Figure 2:**
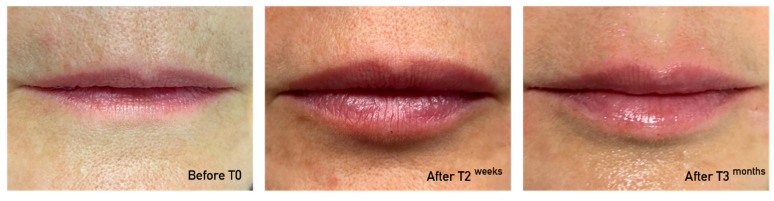
Patient photos before and immediately after injection on day 0.

**Figure 3:**
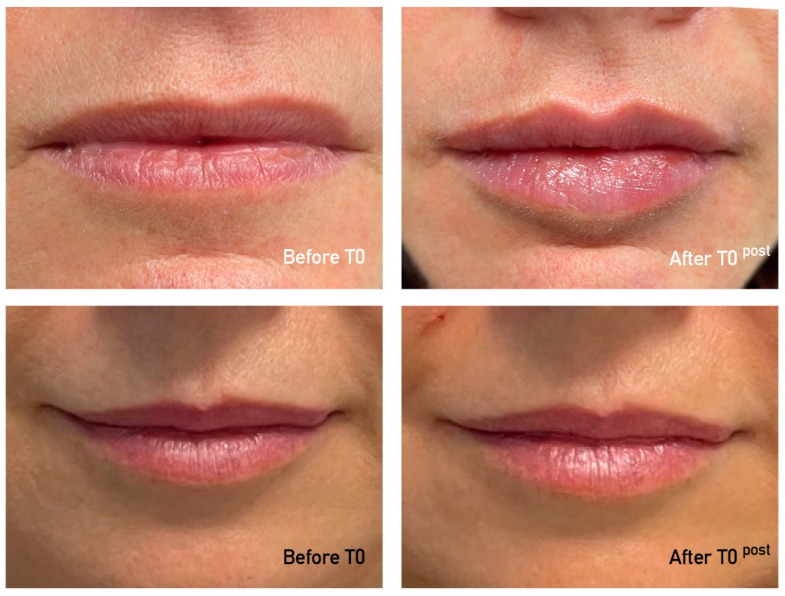
A representative photo series of a patient on day 0 before injection, 2 weeks post-injection, and 3 months post-injection.

Between June 2022 and October 2022, a total of 13 subjects (all Caucasian and women), with an average age of 55.3 ± 8.3 years (range of 42–69 years) had lip enhancement using a dermal HA filler. An average of 1.15 cc of HA was injected per patient (range of 1–2 cc). The representative cases are presented in Figure 2 and Figure 3. It is important to note that not all patients attended the follow-up visits at 3 months (8 out of 13 patients) and 6 months (5 out of 13 patients).

### 3.2. Assessments

**Figure 4:**
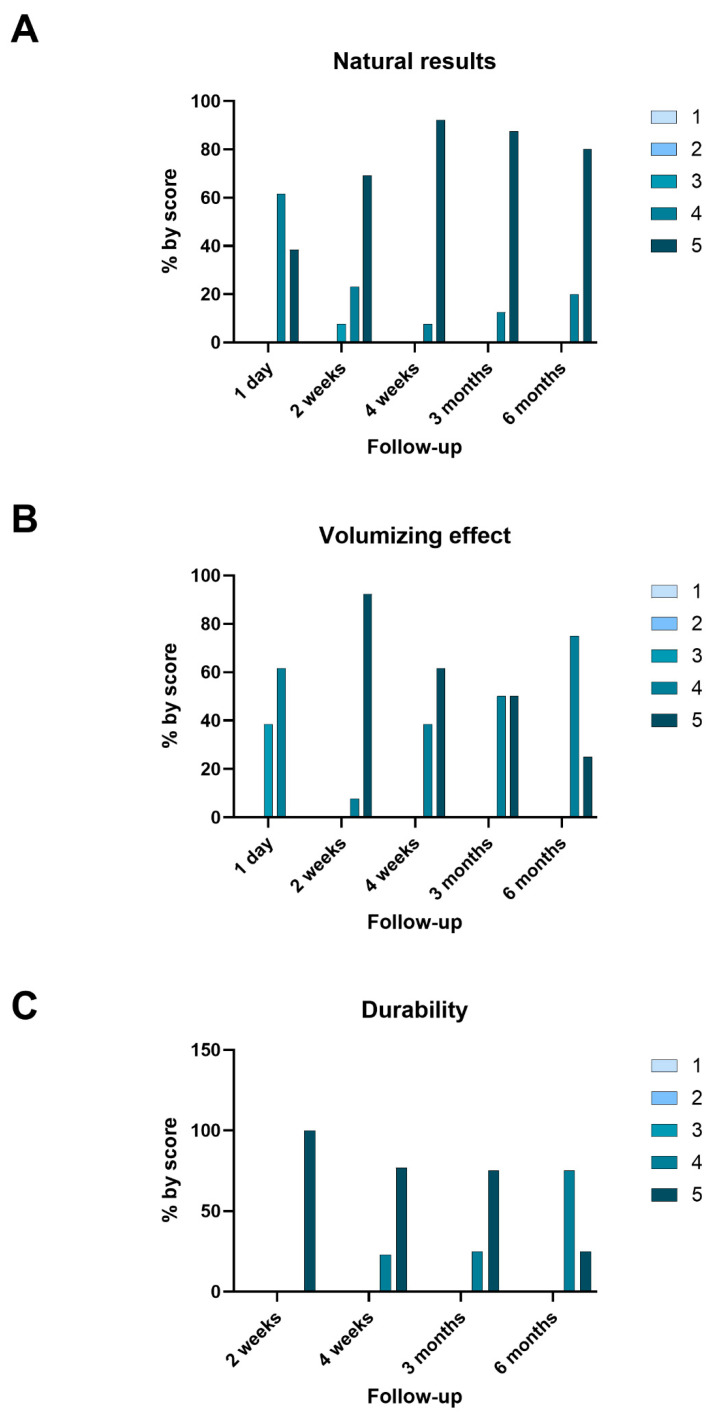
Percentage (%) of patients per score. Results from day 1 post-injection to 6 months post-injection assessed on five-point scale. Natural appearance (**A**), volumizing effect (**B**), and durability (**C**) were evaluated by investigator.

The syringe force was evaluated by the investigator during each procedure using a scale of 1 to 5 (ranging from minimum to maximum) with an average score of 3.2 ± 0.4. The results from day 1 post-injection to 6 months post-injection were evaluated on a five-point scale, as depicted in Figure 4. The evaluation covered natural appearance (Figure 4A), volumizing effect (Figure 4B), and durability (Figure 4C), with each category being assessed by the investigator. The analysis demonstrates that all categories consistently achieved high scores across the entire follow-up period.

A significant difference in natural appearance was found across the follow-up periods (*p* = 0.036). Specifically, the natural appearance scores significantly improved between day 1 and the later follow-up periods, with the greatest improvements being noted between day 1 and week 4 (*p* < 0.05) and 6 months after treatment (*p* < 0.05). No significant differences were observed between the later time points, suggesting that the natural appearance of the results stabilized after 4 weeks.

The volumizing effect showed a statistically significant difference across the follow-up time points (*p* = 1.21 × 10^−5^). The volumizing effect scores improved significantly between 1 day and 2 weeks (*p* < 0.05), 1 day and 4 weeks (*p* < 0.05), and 1 day and 3 months (*p* < 0.05), indicating an increase in the volumizing effect over time. No significant differences were observed between the later time points (4 weeks, 3 months, and 6 months), suggesting that the volumizing effect stabilized after the 4-week mark (*p* < 0.05). These results demonstrate a clear increasing trend in the volumizing effect in the weeks following the procedure, with the effect plateauing after 4 weeks.

The durability scores also showed a statistically significant difference across these time points (*p* = 6.75 × 10^−5^). Significant differences were observed between specific time points. Notably, the durability scores significantly decreased between 1 day and 3 months (*p* < 0.05) and between 2 weeks and 3 months (*p* < 0.05), indicating that the perceived durability of the results reduced over time. No significant differences were observed between the earlier time points (1 day, 2 weeks, and 4 weeks), suggesting stability in perceived durability up to the 4-week mark, after which a gradual decrease was noted by the 3-month follow-up.

**Figure 5:**
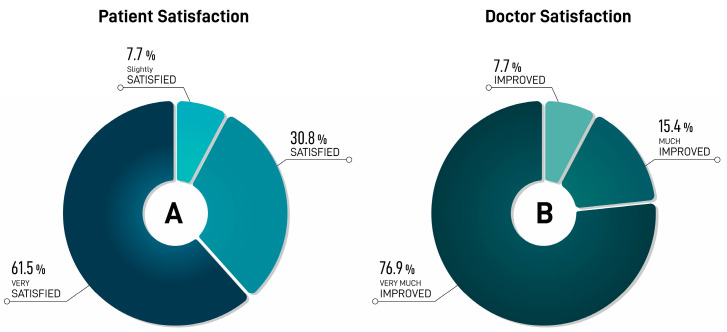
Treatment satisfaction. (**A**) Patient satisfaction levels were evaluated using Likert scale. (**B**) Score results were obtained from physician after completion of GAIS (Global Aesthetic Improvement Scale) questionnaire after procedure.

All of the patients (100%) demonstrated an improvement (“very much improved”, “much improved”, and “improved”) throughout the study. The combined proportion of patients who judged themselves to be “very much improved” and “much improved” was 92.3% (Table 1 and Figure 5A).

The physician (100%) rated the treatment as having some level of improvement (“very much improved”, “much improved”, and “improved”). Only one patient (7.7%) showed an improvement in esthetic results from the initial condition, but a touch-up or re-treatment was considered to further enhance the results (Table 1 and Figure 5B).

### 3.3. Safety Results

The only AEs observed after the injections in all of the patients were swelling and pain.

All patients reported some discomfort during the injection. The patients rated their pain with an average score of 3 ± 0.3, indicating a moderate level of discomfort on a scale with a maximum score of 5. Three patients required a local anesthesia injection (nerve block with lidocaine) due to poorly controlled pain.

Swelling was reported as follows: 100% experienced inflammation within the first 24 h, 61.5% had swelling at 48 h (8 out of 13), and 23.1% (3 out of 13) at 72 h. The physician assessed swelling with an average medium score of 3.5 ± 0.5, also on a scale with a maximum score of 5.

Swelling diminished within the first 7 days after the procedure in all patients. No other AEs were observed by the treating physician during the subsequent follow-up visits or reported by the patients. No severe AEs were reported. None of the patients experienced tenderness, infection, or the Tyndall effect throughout the study period.

## 4. Discussion

This retrospective observational study was conducted to analyze the efficacy and safety of an HA dermal filler treatment for lip augmentation and contouring. To accomplish this, data from 13 participants were retrieved from the database of a Spanish esthetic clinic. This study evaluated the natural appearance, durability, and volumizing effect of the procedure over several follow-up periods, though it is important to note that some patients were lost to follow-up during the later visits. Despite this, the results provide valuable insights. The natural appearance of the results significantly improved over time (*p* < 0.05), particularly between day 1 and week 4, and remained stable through to the 6-month follow-up (Figure 4A). This suggests that the filler becomes better integrated into the tissue over time, enhancing the natural look of the results. The volumizing effect followed a similar trend, with significant improvements being noted between day 1 and week 2 and reaching an optimal level by week 4, after which the volume remained stable for up to 6 months (Figure 4B). This indicates a sustained volume enhancement post-procedure. However, a slight decrease in durability was observed between 4 weeks and 3 months, with significant differences being noted between these time points (Figure 4C). While durability remained stable during the early follow-up, this gradual decline aligned with the expected absorption of the filler material.

The post-treatment assessments by both the patients and doctors indicated a significant rate of improvement. Most of the patients (92.3%) rated the results as “very much improved” and “much improved” (Figure 5A). The physician assessed the treatment outcomes using the GAIS and categorized them as showing varying degrees of enhancement, ranging from “very much improved” to “much improved” and “improved” in all of the cases (Figure 5B). Comparable findings have been documented in existing research. The meta-analysis conducted by Czumbel et al. revealed that using injectable HA for lip augmentation is an effective way to enhance lip volume for at least six months post-treatment [22]. It is important to recognize that patient and physician satisfaction with esthetic treatments is strongly influenced by cultural perceptions of beauty [23]. A comprehensive patient-specific approach must integrate factors such as physiological age and chronological age, ethnically associated facial morphotypes, and culturally determined esthetic ideals related to gender [23]. A study by Hernandez et al. demonstrated that, although fuller lips are typically regarded as more attractive, excessive augmentation in individuals with naturally voluminous lips led to a reduction in perceived attractiveness. This implies the existence of a threshold for filler volume, beyond which esthetic outcomes may deteriorate [10]. However, this study did not account for variations across different ethnicities.

This study observed only short-term AEs, such as redness, swelling, and pain, which are typical for the treatment type and detailed in the product’s Instructions for Use (IFUs). These AEs were managed and resolved within a few days. Studies in the literature focused on the safety of several types of dermal fillers localized in the nasolabial fold area. These studies in general showed that most common AEs are mild, reversible, and injection-related [24]. The most common AEs included lumpiness, tenderness, swelling, bruising, pain, and redness [22,25]. Our study found injection site pain and swelling to be the main complaints after receiving HA dermal filler, as has been described in other studies [22]. This may be because the nasolabial fold and lips are notably delicate due to the facial artery that traces its path, which can explain the observed AEs. Additionally, the appearance of AEs in this region does not seem to be related to the HA filler composition (monophasic HA versus biphasic HA fillers) [24].

HA dermal fillers offer several advantages over other dermal fillers, such as PLLA and calcium CaHA, particularly for lip augmentation. One key benefit of HA fillers is their high biocompatibility, which results in a lower risk of adverse reactions, including granuloma formation, a complication more frequently associated with fillers like PLLA and CaHA [26]. Additionally, HA fillers provide the unique advantage of reversibility. In cases of overcorrection or complications, HA can be effectively dissolved with hyaluronidase, offering a level of safety and control that is not possible with PLLA or CaHA, which are not as easily corrected once injected [27,28]. These factors make HA fillers a preferred option for lip augmentation in clinical practice. This study has several limitations that should be acknowledged. First, the sample size is relatively small, as this study was designed as a case series. While the results provide valuable preliminary data on the safety and efficacy of the lip augmentation procedure, the limited sample size restricts the ability to detect rare or unusual adverse events. A larger sample size would be necessary to comprehensively evaluate the full spectrum of potential side effects and to provide a more robust safety profile. Additionally, the homogeneity of the patient population, consisting of the exclusively of females without comorbidities or parafunctions, limits the generalizability of the findings to a broader population. Furthermore, the short duration of this study restricts the ability to assess the long-term safety and efficacy of the treatment. Future research should aim to explore the procedure’s outcomes over an extended follow-up period to gain a better understanding of its durability and potential delayed side effects. Moreover, the absence of objective outcome quantification is another limitation. Due to the unavailability of suitable technologies for clinical application at the time of the study, we relied on clinical observations. Future studies incorporating advanced quantification technologies will be critical for a more precise evaluation of the results.

Nevertheless, the treatment outcomes were evaluated using two esthetic improvement scales: the Likert scale and the GAIS. The AEs noted were anticipated and consisted solely of temporary, injection site-specific reactions of mild severity, such as erythema and localized swelling, all of which resolved on their own within two weeks. No severe AEs, such as allergic or hypersensitivity reactions, infections, granulomas, skin necrosis, or the Tyndall effect, were observed.

## 5. Conclusions

This retrospective case series demonstrates that lip augmentation and contouring with Genefill Soft Fill^®^ HAfiller is a safe and effective procedure. This study showed consistent improvements in natural appearance, volumizing effect, and durability over the follow-up period, with most patients and physicians reporting high satisfaction levels. The majority of patients rated their esthetic outcomes as “very much improved” or “much improved”, with a stable volumizing effect being observed after four weeks and lasting for up to six months. The safety profile was favorable, with only mild, transient adverse events such as swelling and moderate pain, which resolved within a few days. No severe adverse events were observed.

Future studies involving larger, more diverse populations and longer follow-up periods are necessary to provide a more comprehensive assessment of the efficacy and safety of HA fillers for lip augmentation.

## Figures and Tables

**Table 1:** Patient and doctor satisfaction outcomes. GAIS: Global Aesthetic Improvement Scale.

Patient ID	Postoperative Satisfaction Score	
	Patient (Likert Scale)	Doctor (GAIS)
1	E	2
2	E	1
3	C	3
4	E	1
5	E	1
6	D	1
7	E	1
8	E	1
9	D	2
10	E	1
11	D	1
12	D	1
13	E	1

## Data Availability

The raw data supporting the conclusions of this article will be made available by the authors on request.
